# About value systems and religious orientations among family sobriety club members

**DOI:** 10.1192/j.eurpsy.2025.2188

**Published:** 2025-08-26

**Authors:** A. Magay, Z. Zoricic, A. Baburin, A. Nemtsev

**Affiliations:** 1 Mental Health Research Centre, Moscow, Russian Federation; 2 University Hospital “Sestre Milosrdnice”, Zagreb, Croatia; 3 Tomsk State University, Tomsk, Russian Federation

## Abstract

**Introduction:**

For over 50 years, there has been based on Vladimir Hudolin method a family preventive program for the addicted of psychoactive substances and actions (Zoricic 2006; 42(1) 35). Family therapeutic communities (family sobriety clubs) exist in many countries in Europe and the world, in Russia there are over 50 clubs. In Russian clubs, a change in the lifestyle of rehabilitants is accompanied by a transformation of value systems and semantic formations into traditions of religious worldview.

**Objectives:**

To study the dynamics of changes in value systems and religious beliefs among family sobriety club members during rehabilitation, which includes a spiritually-oriented component.

**Methods:**

50 Russian patients with alcohol addiction who participated in rehabilitation in a community setting were examined: 25 (“participants” group) - with remission from several months to two years, 25 (“leaders”) – with remission over two years were also the heads of family sobriety clubs. There were used psychometrical (Schwartz Value Survey – SVS, Religions Orientation Scale - ROS) and statistical methods.

**Results:**

Leading values among “participants” are “kindness”, “safety” and “conformality”, the most significant values among “leaders” are “kindness”, “traditions” and “universalism” (р<0,05). Benchmarking: *self-transcedence* values prevail among “leaders”, high levels of *self-raising* among “participants”. Analysis of variance (ANOVA): increasing the significance of values of *kindness* (p = 0.025) and *tradition* (p = 0.009) at the level of ideals, *tradition* (p = 0.0003) and *safety* (p = 0.04) at the level of priorities at remote stages of rehabilitation. The cohesion of values among “leaders” is higher than among “participants” (*conformity* (p = 0.03) and *kindness* (p = 0.09)). Over time a detailing of spiritual ideas takes place, rehabilitants identify themselves as believers and aim to change their lifestyle according to religious Faith values. According to ROS, there is a more meaningful religiosity among “leaders” than among “participants,” the predominance of internal religiosity over external religiosity at long stages of assistance.

**Conclusions:**

Change in value orientations correlates with the duration of rehabilitation, their greater cohesion is noted, values are more consistent with the normative for the population as a whole. Religious idias become more harmonious, a transformation of dysfunctional manifestations of external religiosity into adaptive forms of internal religiosity is noted, which is associated with the increase of overall meaningfulness of life.

**Disclosure of Interest:**

None Declared

